# Media Competition Implementation for the Massachusetts Childhood Obesity Research Demonstration Study (MA-CORD): Adoption and Reach

**DOI:** 10.3390/ijerph13040403

**Published:** 2016-04-05

**Authors:** Shaniece Criss, Lilian Cheung, Catherine Giles, Steven Gortmaker, Kasisomayajula Viswanath, Jo-Ann Kwass, Kirsten Davison

**Affiliations:** 1 Department of Social and Behavioral Sciences, Harvard T.H. Chan School of Public Health, 677 Huntington Avenue, Kresge Building, Boston, MA 02115, USA; cgiles@hsph.harvard.edu (C.G.); sgortmak@hsph.harvard.edu (S.G.); vish_viswanath@dfci.harvard.edu (K.V.); kdavison@hsph.harvard.edu (K.D.); 2 Department of Nutrition, Harvard T.H. Chan School of Public Health, 655 Huntington Avenue, Boston, MA 02115, USA; lcheung@hsph.harvard.edu; 3 Dana-Farber Cancer Institute, 450 Brookline Avenue, Boston, MA 02215, USA; 4 Massachusetts Department of Public Health, Bureau of Community Health and Prevention, 250 Washington Street, Boston, MA 02108, USA; jo-ann.kwass@state.ma.us

**Keywords:** media competition, elementary school, implementation, reach, adoption, childhood obesity

## Abstract

The Massachusetts Childhood Obesity Research Demonstration Study (MA-CORD) was a multi-level, multi-sector community intervention with a media competition component to provide an overarching synergy and promote awareness of target behaviors to reduce childhood obesity. Students participating in the media competition were tasked with developing videos, song/rap lyrics, and artwork that reflected the goals. The aim of this study is to document the process used to develop and implement the media competition along with its reach and adoption. An adapted version of Neta and colleagues’ 2015 framework on dissemination and implementation was used to summarize the process by which the media competition was developed and implemented. Adoption was defined by whether eligible schools or afterschool programs decided to implement the media competition. Reach was defined by student participation rates within schools/programs and the number of votes cast for the finalists on the coalition website and students’ paper ballots. A total of 595 students participated in the media competition from 18 school and afterschool programs in two communities. Adoption of the media competitions ranged from 22% to 100% in programs and reach ranged from 3% to 33% of the student population. The documentation of the implementation should contribute to the replication of the media competition.

## 1. Introduction

Obesity is an urgent public health concern in the United States, particularly among children. Data from the 2011 to 2012 National Health and Nutrition Examination Survey (NHANES) reveal that 17% of all children and adolescents in the U.S. are obese [1]. Excess weight in childhood is associated with early indicators of chronic disease and increased risk of overweight and obesity during adolescence and adulthood; and elevated risk of chronic diseases and premature death as an adult [2]. Multi-sector community interventions, which engage stakeholders across multiple sectors such as early care and education, schools, afterschool programs and health centers, in childhood obesity interventions, are recommended given the complex etiology of obesity which encompasses, biological, psychosocial, and behavioral factors [3]. 

Although still in their infancy, multi-level community interventions to reduce childhood obesity have been effective in reducing body mass index (BMI) [4,5]. With the complexities of these interventions, there can be difficulties with translation from research to practice [6]. Therefore, an iterative process is required through replication and triangulation of data from multiple study designs to guide the translation of complex multi-level health interventions into real-world settings [7]. Dissemination of an intervention is not an end in itself, but the successful integration and implementation by the end user are key indicators for success in practice-based settings [8]. 

### MA-CORD Overview

The Massachusetts Childhood Obesity Research Demonstration Study (MA-CORD) is a multi-level, multi-sector community intervention to prevent and reduce childhood obesity among low-income children, aged 2–12 years in two communities in Massachusetts (MA). Consistent with the Obesity Chronic Care Model [9], MA-CORD incorporates evidence-based interventions in each sector including healthcare, early care and education, the Special Supplemental Nutrition Program for Women, Infants, and Children (WIC), schools, afterschool programs, and the broader community (known as *Mass in Motion Kids* in intervention communities). The MA-CORD intervention design is outlined in detail by Taveras *et al.* [10], and the evaluation plan and baseline data are presented in Davison *et al.* [11]. MA-CORD was funded by the Centers for Disease Control and Prevention as part of a comprehensive approach in several cities across the U.S. to address childhood obesity [12].

This study focuses on a media competition implemented with students in public elementary and middle school and afterschool programs in the MA-CORD communities. Studies indicate that interventions that integrate a communications campaign, such as media arts competitions, can improve student awareness and behavior change [13,14]. A youth empowerment approach that includes youth developing and executing media campaigns increases children’s self-efficacy and a sense of responsibility through the production of knowledge that impacts policy and action in their communities [15]. This type of intervention has the potential to contribute to positive outcomes. 

The goal of the media competition was to provide an overarching synergy for MA-CORD and promote awareness of the target behaviors which included: (1) Switch from sugary drinks (like soda, sports drinks, and fruit drinks) to water; (2) Watch no more than two hours of screen time per day (includes TV, smartphones, and hand-held video games); (3) Get at least one hour of physical activity (including active play) per day; (4) Replace sugary, salty, fried, and fast food with fruits and vegetables; and (5) Sleep at least 10 h (2–5 year olds) or 11 h (6–12 year olds) per day. In particular, students were tasked with developing videos, song/rap lyrics, and artwork that reflected the question “How can you be a *Mass in Motion Kid*?” by addressing the goals. Media competitions are important components of interventions, yet many studies do not focus on the detailed planning and implementation process for replication in school and afterschool programs. Therefore, in this study we document the process used to develop and implement the media competition and present measures of its adoption and reach. 

## 2. Methods

### 2.1. Empowerment Approach

The media competition adopted an empowerment approach on two levels. First, the competition as a whole was developed by school and community representatives in collaboration with the researchers. Second, the competition promoted students’ active and meaningful engagement with the development and creation of their media competition entries to effect change in their community [16]. Incorporating an empowerment approach was particularly salient in this multi-sector intervention because community coalitions partnered with this study, which provided an opportunity for students’ work to reach beyond the school or afterschool program.

### 2.2. Theoretical Framework

**Figure 1:**
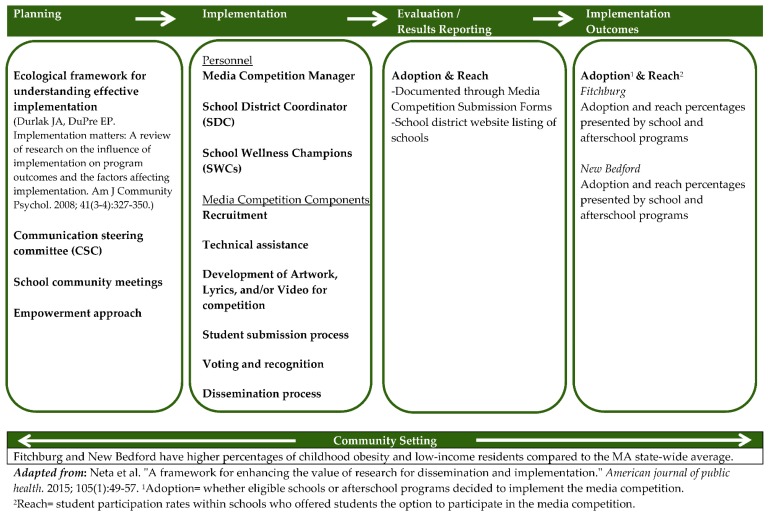
Media competition implementation process.

Detailed documentation of the process to implement an intervention can facilitate its replication in other communities [17]. An adapted version of Neta and colleagues’ 2015 framework on dissemination and implementation is used to summarize the process by which the media competition was developed and implemented [17]. Figure 1 shows the Media Competition Implementation Process, which includes planning, implementation, evaluation/results reporting, and implementation outcomes.

### 2.3. Community Setting

Understanding the community setting, which is shown at the base of the model in Figure 1, is essential for successful intervention adoption [18]. For MA-CORD, the community context was a cross-cutting factor that affected each aspect of implementation. The media competition was implemented in Fitchburg and New Bedford, Massachusetts. Table 1 lists the characteristics of each community. Fitchburg has 40,514 residents with 6 public schools serving kindergarten through 8th grade, and New Bedford has 95,502 residents with 23 public schools serving kindergarten through 8th grade, including 21 elementary schools and 2 middle schools. Across both communities, non-Hispanic white residents are the majority population (68% of residents) and Hispanics (18%–22%) are the largest minority group. Both communities have a higher percentage of low-income residents and children classified as overweight or obese compared to the MA state-wide average.

### 2.4. Implementation

**Figure 2:**
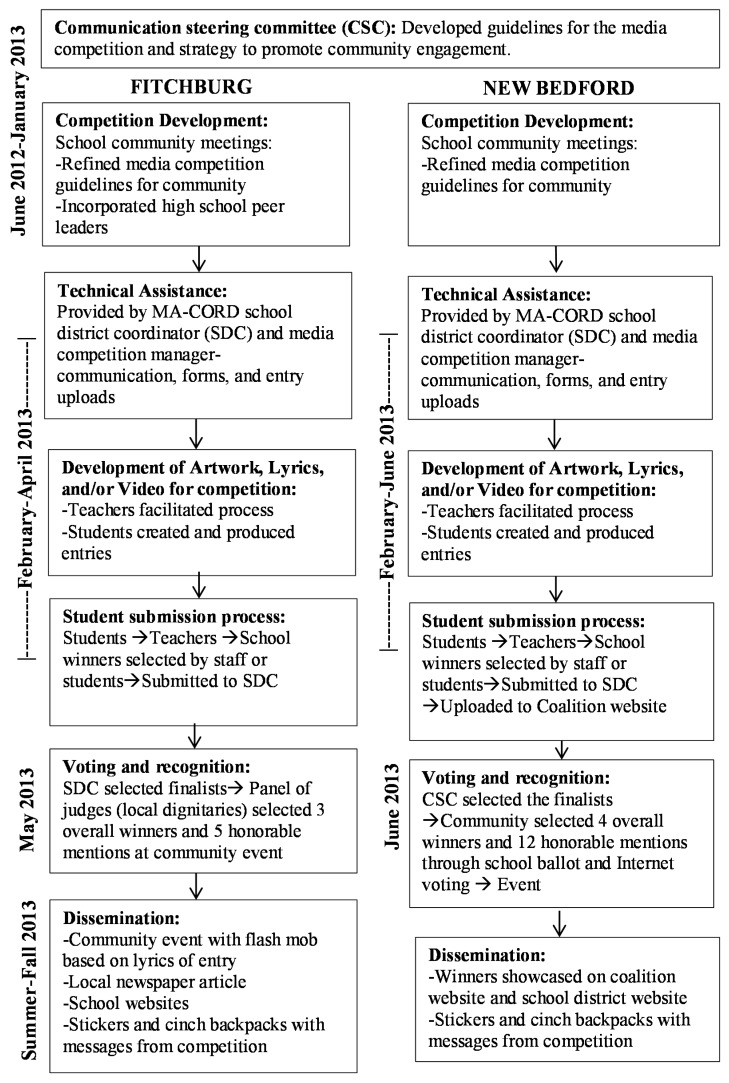
Media competition intervention flowchart.

Figure 2 illustrates the Media Competition Intervention Flowchart. It focuses on the competition timeline highlighting competition development, technical assistance, development of the artwork, lyrics, and video entries, voting and recognition, and dissemination.

#### 2.4.1. Communication Steering Committee (CSC)

The research team initiated a Communication Steering Committee (CSC) to guide the development of the media competition and further communication initiatives for the broader MA-CORD intervention. The committee consisted of teachers, nurses, and coalition members from each community, and representatives from the Massachusetts Department of Public Health and Harvard T.H. Chan School of Public Health. The CSC oversaw the media competition process and provided solutions concerning research and implementation issues. One main decision point was offering the students three types of submissions options (*i.e.*, video, lyrics, and artwork) instead of one option (e.g., poster). The full CSC met twice and a smaller subset met regularly at the weekly MA-CORD meetings during the development and implementation of the media competition.

#### 2.4.2. School Community Meetings

Three meetings with school and community representatives were held in each community to refine components of the media competition including the competition guidelines, promotion of the competition, the voting process, and the prizes. Specialty (e.g., health, art, physical education) and classroom teachers, cafeteria managers, school nurses, and coalition members (including a youth group in Fitchburg) participated in the school community meetings. Based on all these meetings, the media competition manager (from the research team) adapted the media competition for each community to accommodate school district regulations and community preferences.

#### 2.4.3. Technical Assistance

Key personnel included the media competition manager, a school district coordinator from each community, and school wellness champions from each school. The media competition manager coordinated the planning, delivery, and evaluation. The school district coordinators, which were school nurses, coordinated the compilation of entries within schools and were the main liaison between the study team and school personnel. They were instrumental in providing technical assistance with the submission process by scanning parent permission forms and uploading entries on SchoolTube, which is similar to YouTube with the addition of teacher moderation. Each school also identified one school wellness champion, typically a teacher or nurse. They received training and technical assistance about MA-CORD school intervention components and coordinated efforts within their school. The school district coordinators worked with the school wellness champions to recruit teachers to offer the competition to their students through email, fliers, and on-site visits. Technical assistance for the media competition was focused on teachers; they could then assist their students.

#### 2.4.4. Guidelines

Each teacher received detailed guidelines for the media competition, including the requirements for each submission type. School and afterschool programs had the latitude to conduct the competition to best fit their schedules. The guidelines requested that schools submit their top three selections in each category: artwork, lyrics, and video. Videos and song/lyrics could be filmed using a smartphone, tablet, computer camera, camera with video option, or video camera and had to be under 2 min long. The artwork had to be drawn on a piece of paper (any size and color accepted) and with any medium (e.g., colored pencils, markers, crayons, paint, *etc.*). Students were allowed to use school and personal equipment and materials. Students could work individually or in groups. Several schools had within-school competitions where students voted for their favorite entries.

#### 2.4.5. Voting

Once students submitted their entries to their teachers, the school selected entries to submit to the district-level through teacher, staff, or student vote. Parent/Guardian Media Release Forms were required for each student who submitted their entry to the district-level competition. At the district level in Fitchburg, the school district coordinator selected the finalists and a panel of local dignitaries (e.g., mayor, state legislator) selected three overall winners and five honorable mentions. In New Bedford, the Communication Steering Committee selected the finalists. The finalist entries were posted on the New Bedford coalition website. Community members could vote through the website and participating schools’ students could vote by submitting a paper ballot. Four overall winners and 12 honorable mentions were selected and celebrated at a community event. 

#### 2.4.6. Recognition

For each winning entry, the school, teacher, and student received a prize. Fitchburg received gift certificates for educational materials, and New Bedford received gift cards from Amazon. In addition, the dissemination process consisted of showcasing the winning entries in the communities, including coverage on websites (e.g., coalition, school district), a story in the local newspaper, a community event with a flash mob based on lyrics of entry, and distribution of stickers and cinch backpacks (*i.e.*, drawstring backpacks) with messages from the competition.

### 2.5. Participants

All public elementary and middle schools and affiliated school-based afterschool programs in the two MA-CORD intervention communities were eligible to participate in the media competition. In participating school and afterschool programs, students in kindergarten through 8th grade were eligible to submit a media competition entry. 

### 2.6. Measures: Reach and Adoption

In this study, reach was defined by student participation rates within schools/afterschool programs that offered students the option to participate in the media competition. In New Bedford, reach was also measured through the number of votes cast for the finalists on the coalition website and participating schools’ students’ paper ballots. For both communities, adoption was defined by whether eligible schools or afterschool programs decided to implement the media competition [19].

### 2.7. Data Collection and Analysis

To document reach and adoption of the media competition across the two communities, we collected process data through the competition submission forms. Teachers in each participating site submitted a form for each entry (*i.e.*, for each song, poster, or video) which collected information on the school/afterschool program, the name of the entry and category it was submitted to, and the number of students who participated in developing the entry and the role of each student (e.g., actor, writer). In addition, we confirmed the number of schools in each community through their school district website. Data analysis consisted of calculating the adoption (number of schools/afterschool programs divided by total number of programs) at the district level for schools and afterschool programs and the reach (number participants divided by number of all students in program) of the competition within each school or afterschool program.

## 3. Results

### 3.1. Participation Levels

Table 2 describes the media competition’s reach (participation levels) by school and afterschool programs in each community. A total of 595 students participated in the media competition from 18 school and afterschool programs. All entries were submitted in English.

### 3.2. Adoption

In Fitchburg, six schools (three elementary schools, three middle schools) and one afterschool program in a middle school had 10 teachers submit 38 student entries (10 videos, 11 songs/raps, 17 posters). The adoption in Fitchburg (*i.e.*, whether eligible schools or afterschool programs decided to implement the media competition) was 100% for schools (six out of six eligible schools) and 17% for afterschool programs (one out of six eligible afterschool programs, measured by staff member participating in a MA-CORD learning community). In New Bedford, nine schools (eight elementary schools, one middle school) and two elementary afterschool programs had 20 teachers submit 58 entries (10 videos, 10 songs/raps, 38 posters). The adoption in New Bedford was 39% for schools (9 out of 23 eligible schools) and 22% for afterschool programs (two out of nine eligible afterschool programs).

### 3.3. Reach

The reach (% student participation) of the media competition within schools and after school programs ranged from <1% to 33%. Schools/after school programs were classified as demonstrating low reach (<1%–3%), moderate reach [>3% with <100 participants; based on having a number of students that could connote at least the participation of one full class (e.g., at least 20 students in a school setting)], and high reach (>3% with ≥100 participants). In Fitchburg, six out of seven schools demonstrated moderate reach and one demonstrated high reach. Average reach in Fitchburg (*i.e.*, student participation rates within schools who offered students the option to participate in the media competition) was 10% for schools and 33% for the one afterschool program with a total of 377 student participants. In New Bedford out of 11 programs, seven schools demonstrated low reach, three had moderate reach, and one exhibited high reach. Average reach in New Bedford was 3% for schools and 9% for afterschool programs with a total of 218 student participants. In addition, 1400 people voted on the district-level finalists to select the winners through school ballot (850 students) and Internet on the coalition website (550 community members).

## 4. Discussion

This study outlines the planning, implementation, adoption, and reach of the media competition to provide much needed “how-to” documentation for end users. The development consisted of a participatory process with significant community input, and the implementation was led and facilitated by community members with technical assistance provided by the research team. Eighteen schools/afterschool programs participated and 595 students submitted entries. School adoption rates were higher than the afterschool adoption rates. Each community had one school with high reach (*i.e.*, over 100 students submitting an entry). Fitchburg had more programs with moderate reach than New Bedford, and New Bedford had more students and community members engaged with the process through 1400 people voting.

The MA-CORD media competition demonstrated a 100% school adoption rate in Fitchburg and 39% school adoption rate in New Bedford, and had up to a 30% reach of students in schools in Fitchburg and 13% in New Bedford. About one to three teachers submitted an entry form per participating school. It is difficult to identify appropriate interventions against which to compare reach and adoption rates of the MA-CORD media competition. Among school-based interventions, adoption and reach can be measured at the level of the school, class, and/or teacher. There can also be great variation in the interventions themselves, the extent to which intervention activities are mandated *versus* elective, and whether implementation is led by researchers *versus* the school or community. All of these factors can affect adoption and reach rates. In this instance, the MA-CORD media competition was a supplemental activity in which teachers and students had the option to complete outside of class time. Teachers had to dedicate instructional time in order for students to have time to prepare entries during the school day or organize opportunities outside of the school day. In comparison, National Football League (NFL) Play 60 FITNESSGRAM^®^ Partnership Project is an optional in-school nutrition and physical activity community intervention, which had about a 20% teacher adoption rate in teachers participating in programming [20]. One programming component is the “Touchdown Dance Challenge” and has students submit a touchdown dance through a still image, a video, or slideshow with a NFL player teaching the national winner’s dance to their school [21]. The level that we measured adoption for our study (school/afterschool level) had higher levels of adoption than teacher adoption rates in Play 60 programming, but Play 60 is a national program with over 16 million participating students [22]. 

The researchers identified key lessons learned from documenting the variation in reach and adoption rates across schools and communities from submission forms and interactions with the key stakeholders throughout the process. An accompanying article in this supplement examines other factors that explain the variation of the media competition reach through 54 key stakeholder interviews in order to improve implementation. On the community level, both school districts supported the MA-CORD study initiatives, but they did not require schools to participate in the media competition. In Fitchburg, all eligible schools participated. That school district (six schools serving kindergarten to 8th grade) was smaller than New Bedford’s school district (23 schools serving kindergarten to 8th grade), so it was easier for the school district coordinator to communicate on a personal level with school staff. School districts should ensure there is a structure within their schools to provide students time to work on their entries for greater reach by incorporating it within a class lesson (e.g., health, art, music, language, *etc.*). On the school level, the wellness champions had varying levels of participation. If the wellness champion was vested in the media competition and did not have other competing priorities, then student reach seemed to be higher. In addition, afterschool settings are growing as settings for childhood obesity prevention [23,24,25,26]; therefore, the school-based afterschool programs could have been a site for more participation. Afterschool programs were not assigned a wellness champion so some afterschool staff may not have been motivated to initiate the program or provide instructional and technical support. Another lesson learned is that some teachers and students were not clear about the benefits of this new media competition. After the roll-out, many of the teachers reported that they thought more schools, teachers, and students would participate in following years. If the media competition was an annual event, the community school meetings should be continued to advise the implementers on the contextual factors for each period. Most of the challenges with implementation could have been avoided with even more engagement and consultation with the members of the school community members. For instance, it is important to understand the budget regulations and allocations for prizes for students, as well as, the policy of students presenting their work on the Internet. 

### Strengths and Limitations

We did not test the effectiveness of the media competition independent of the other intervention activities of MA-CORD, yet we were able to collect implementation data to inform future iterations of a similar media competition. Impacts of the MA-CORD Study will be reported in forthcoming studies. In future studies, it would be helpful to collect other process-related measures such as fidelity, which is the degree an intervention was delivered as intended [27]. Strengths of this study are the substantial involvement of the communities and school personnel in the development and implementation process. Another strength is that each school and teacher was able to adapt the media competition for their students. In order to collect the adaptations, we captured information on the implementation process for each school on the submission form required for the district level competition, which we would recommend as a technique for other studies because the teachers provided relatively complete data. Furthermore, members from our study team conducted qualitative interviews of teachers to understand more about effective implementation [19] of the media competition. 

## 5. Conclusions

The adapted Neta *et al.* framework [17] provided an efficient guide to document the entire implementation process to support replication of the MA-CORD media competition. This study highlights the importance of truly engaging key stakeholders early and often for implementing community interventions, as well as practical logistical aspects of developing a media competition within school and afterschool programs. Researchers should continue to measure adoption and reach as an indicator of dissemination to help explain implementation *versus* intervention effects.

## Figures and Tables

**Table 1:** Characteristics of the community setting for the media competition.

Socio-Demographic Characteristics	Massachusetts	Fitchburg	New Bedford
Total population ^1^	6,436,940	40,514	94,502
% White (any race) ^1^	76.1	68.2	67.9
% Black or African American (any race) ^1^	6.0	1.1	5.2
% Hispanic or Latino (any race) ^1^	9.6	21.6	16.7
% of children overweight or obese 2009–2010 ^2^	33.4	46.2	37.2
Average per capita income ^3^	$35,485	$22,949	$21,343
% Families with children whose incomes are less 100% or more of the Federal Poverty Level ^3^	12.0	23.5	27.1
# of public schools serving students kindergarten through 8th grade 2012–2013 ^4^	~1500	6	23

^1^ 2010 Census; ^2^ Massachusetts Department of Public Health; ^3^ 2008–2012 American Community Survey 5-year estimates; ^4^ Massachusetts Department of Education.

**Table 2:** Media Competition Participation Levels by School and Afterschool Programs in each community.

School Type	Participation		Reach (Student Participation) Level	Grade Level
	# of Students	Reach (% Student Participation)		
Fitchburg				
Elementary School *	40	6%	Moderate	2nd–4th
Elementary School *	192	30%	High	3rd–4th
Elementary School *	25	4%	Moderate	4th
Middle School *	25	4%	Moderate	5th–7th
Middle School *	50	8%	Moderate	5th–7th
Middle School (MI)	30	6%	Moderate	5th–6th, 8th
Middle Afterschool *	15	33%	Moderate	5th–7th
*Community Total:*	377	10% (schools) 33% (afterschool)		
New Bedford				
Elementary School *	38	5%	Moderate	4th–5th
Elementary School (NI)	23	4%	Moderate	3rd–5th
Elementary School *	120	13%	High	4th
Elementary School (NI)	3	<1%	Low	3rd
Elementary School *	11	<1%	Low	5th
Elementary School (NI)	2	<1%	Low	5th
Elementary School (MI)	10	3%	Low	5th
Elementary School (MI)	2	<1%	Low	5th
Middle School (NI)	1	<1%	Low	Not specified
Elementary Afterschool (MI)	3	1%	Low	2nd–3rd
Elementary Afterschool (NI)	5	16%	Moderate	5th
*Community Total:*	218	3% (schools) ^+^ 9% (afterschool)		
*Intervention Total:*	595			

* Media Competition Interview | MI = MA-CORD Interview | NI= No interview; Note: Some programs included interviews from multiple teachers; ~ Participation Level = Low = <1%–3% of student participation in school (School Range: 1–11 students; Afterschool Range: 3 students); Moderate = >3% with <100 participants (School Range: 23–50 students; Afterschool Range: 5–15 students); High: >3% with >100 participants (School Range: 120–192); ^+^ Plus 1400 students and community members voted on the district-level finalists; Note: In New Bedford, six participants (three from elementary schools, one from a middle school, and two from an elementary/middle afterschool program) participated in a MA-CORD interview, but their program did not submit any entries to the competition.
